# Changes in chemical composition and biological activity of essential oil from Thomson navel orange (*Citrus sinensis* L. Osbeck) peel under freezing, convective, vacuum, and microwave drying methods

**DOI:** 10.1002/fsn3.1279

**Published:** 2019-12-04

**Authors:** Reza Farahmandfar, Behraad Tirgarian, Bahare Dehghan, Azeeta Nemati

**Affiliations:** ^1^ Department of Food Science and Technology Sari Agricultural Sciences and Natural Resources University Sari Iran

**Keywords:** antimicrobial, antioxidant, drying, essential oil, Thomson peel

## Abstract

Thomson navel orange peel is a by‐product of citrus processing, which contains high levels of bioactive compounds advantageous to human health, nevertheless due to its high moisture content it is exceedingly perishable. Drying is among the most common preservation methods, which could prolong the plants shelf‐life via reducing their moisture value. Taking this into account, depending on their type and conditions, drying techniques could degrade plant heat‐sensitive metabolites and lead to quality decline. Therefore, the goal of this paper was to investigate the influence of seven drying methods named sun, shade, oven, vacuum oven, microwave, and freeze‐drying with different drying conditions on the physical properties, for example, bulk density and color (L*, a*, b*, Δ*E*, and browning index (BI)) and essential oil characteristics such as extraction yield, chemical composition, antioxidant (total phenolic content (TPC), DPPH, and FRAP essays), and antimicrobial (MIC and MBC) activities of Thomson peel and determine the superior drying procedure. Results showed that freeze‐dried sample had the highest retention of L* (48.54) and b* (49.00) values, lowest BI (216.11) as well as highest EO extraction yield (6.90%), TPC (60.10 GAE/100 g), FRAP (0.52% at 80 mg/ml), and lowest IC50 (5.00 mg/ml), MIC and MBC compared with other drying treatments. Therefore, it could be inferred that freeze‐drying is the most efficient drying approach in respect of preserving both physical and EO attributes of Thomson peel.

## Graphical abstract

In this research, chemical composition and biological activity of Thomson navel orange peel were analyzed under different drying conditions. Various drying methods increased the monoterpene hydrocarbons of essential oil. Freeze‐dried peels had the highest retention of L* and the lowest formation of brown pigments. Freeze‐dried peels is the best method to maintain antioxidant and antimicrobial activity.

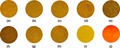

## 1 INTRODUCTION

Orange, *Citrus sinensis* L. of the family *Rutaceae*, is one of the most abundant fruit crops in the world and is well‐received by consumers due to its attractive color, pleasant aroma, and flavor (Juhaimi, Matthäus, Özcan, & Ghafoor, 2016; Matthaus & Özcan, 2012; Smeriglio et al., 2019). Among the most popular orange varieties, Thomson navel (*Citrus sinensis* L. *Osbeck*) is the one that has good economic value and is widely cultivated in countries like Iran, China, India, USA, and Brazil (Faostat, 2018; de la Torre et al., 2019). On a worldwide scale, around 40% of the orange production is utilized in orange juice processing, which generates an enormous amount of wastes (mostly peels) on an average mass of 0.5 kg/kg of raw orange (Smeriglio et al., 2019). Therefore, orange peels as the primary waste have been either discarded which may cause environmental pollutions or used as molasses for animal feed (Gavahian, Chu, & Mousavi Khaneghah, 2019). However, in the outer layer of the orange peels known as flavedo, within a large number of very small glands, essential oils (EOs) are placed (de la Torre et al., 2019). Over the past few years, the biological activities (e.g., antioxidant, anti‐inflammatory, antiaging, antibacterial, antifungal, and anti‐aflatoxigenic activities) of orange peel EOs have been specified (Barreca et al., 2017; Celano et al., 2019; Hasija, Ibrahim, & Wadia, 2015; Kamal, Ashraf, Hussain, Shahzadi, & Chughtai, 2013) which are strongly related to various constituents of these volatile oils including hydrocarbons, alcohols, esters and aldehydes (Geraci, Di Stefano, Di Martino, Schillaci, & Schicchi, 2017). Therefore, the problem of wasted orange peels could be turned into an asset, if potentially marketable proceedings such as EO extraction occurs (Gavahian et al., 2019). Nevertheless, the most critical challenge in regard to orange peels is their high moisture content (75%–90%), which makes them highly perishable with very low storage life (de la Torre et al., 2019). Hence, in order to preserve them for future utilization, their water levels required to diminish. Drying is commonly used to preserve fresh plant materials, and it can be carried out at different temperatures and relative humidity conditions (Samadi, Larijani, Naghdi Badi, & Mehrafarin, 2018). The removal of moisture from plants, primarily retards many of the moisture‐mediated deteriorative reactions and prevents the growth and reproduction of microorganisms (Naidu et al., 2016). Likewise, it has been acknowledged that moisture reduction could cause a remarkable boost in extraction yield of plant's EOs (Franco‐Vega, Ramírez‐Corona, Palou, & López‐Malo, 2016). It is worth noting that dried orange peels could also be employed in food formulation applications such as dairy products, beverages, bakery products, and candy industries (Ghanem, Mihoubi, Kechaou, & Mihoubi, 2012). There are different drying processes such as natural sun drying (SD), shade drying (ShD), oven drying (OD), vacuum oven drying (VOD), microwave drying (MW), and freeze‐drying (FD) (Xing et al., 2017). Each has a different mechanism by transferring different energies at various speeds and times into the product, which would lead to multiple irreversible chemical and biological reactions accompanied by several structural, physical, and mechanical alterations (Xing et al., 2017). Therefore, it is necessary to evaluate the influence of different drying methods on Thomson peels before selecting a desirable method for commercial drying.

So far as we know, there are several studies on the chemical identification and antioxidant abilities of EOs retrieved from fresh Thomson peels (Kamal et al., 2013; Kirbaslar, Kirbaslar, Pozan, & Boz, 2009; Nekoei & Mohammadhosseini, 2014; Njoroge, Phi, & Sawamura, 2009). However, no information is available concerning the effect of various drying methods on the physical characteristics of dried Thomson peel and its EOs quality and quantity. Thus, this work was carried out to determine the possible effect of drying techniques on the physical aspects of dried matter, and consequently on the chemical profile, antioxidant and antibacterial activities of EOs extracted from this valuable by‐product.

## 2 MATERIALS AND METHODS

### 2.1 Materials

The study was carried out in October 2018 from one selected orchards of Amol, Northern Iran (36^◦^46'N and 52^◦^35'E, around 76 m above sea level), and fruits were harvested at commercially mature stage (about 7.5–8.0 months after flowering, fully colored) from approximately 12‐to 15‐year‐old trees on the third week of October. After collection, oranges were brought to a laboratory at Sari Agricultural Sciences and Natural Resources University, Mazandaran province and were rapidly processed on the same day. They were washed under running tap water and patted dry in an attentive manner. The flavedo of fruits was carefully removed with a manual peeler and cut into small pieces. With the intention of preserving their original freshness, collected samples were stored in a refrigerator at 4°C until used in the drying experiments. All chemicals and solvents applied in this research were purchased from Merck (Darmstadt, Germany).

### 2.2 Drying of fresh orange peels

Samples were randomly divided into ten batches each containing 50 g orange peels. One was used for fresh analysis, and the remaining parts were dried by using the following techniques: (a) shade‐dried samples were attained under natural air flow at room temperature (20°C ± 5°C) up to 60 hr; (b) for the purpose of obtaining sun‐dried samples, the plants were left under direct sun/day light at temperatures between 25 and 37°C for 36 hr; (c) conventional oven was applied in a laboratory oven (BM55E, Fan Azma Gostar Co.) at two temperatures of 45 and 60°C for 5 and 4 hr, respectively; (d) a vacuum oven (VS‐1202, Vision Scientific Co. Ltd.) was utilized for VOD at 45°C and 60°C up to 48 and 36 hr, respectively; (e) a microwave oven (MA3884VC, LG Electronics Co.) with a maximum power output of 900 W, which was equipped with a swivel tray plus digital setting for power and time, was applied for drying the samples. The samples were placed in a commercial microwave oven, and drying was done at two different microwave power levels of 360 and 600 W for 35 and 20 min, respectively; (f) the samples were dried by a freeze‐drier (Vaco 2 zirbus) at a temperature of −50°C and pressure of 0.125 mbar till 24 hr for the sake of obtaining freeze‐dried samples. Finally, samples were ground into a fine powder by a Bosch MKM6000 laboratory mill (Bosch Instruments) and passed through a laboratory screen mesh no.16 and stored in bags at −18°C until analyzed. The initial moisture content of fresh Thomson peels was 77.3 ± 0.9% (wet basis), and all samples were dried to reach a constant weight.

### 2.3 Physical properties of dried powders

#### 2.3.1 Bulk density

Bulk density was determined by pouring 5 g of dried Thomson peel powder into an empty 25 ml glass cylinder, and then a gentle tapping of the cylinder was applied until a negligible difference in volume of samples was observed. Bulk density was determined by dividing the weight of sample to its volume and expressed as grams per milliliter (g/ml) (Razavi & Farahmandfar, 2008).

#### 2.3.2 Color measurement

Color alterations in dried Thomson peel samples were analyzed by measuring the parameters of L* a* b* using the IMG‐Pardazesh Cam‐System colorimeter, which was calibrated by a standard calibration plate of a white surface provided by the manufacturer. L* indicates the darkness‐lightness, a* greenness‐redness, and b* blueness‐yellowness of samples. The total color variation index (Δ*E*) was determined by Equation (1), where the subscript “0” in equation refers to the color of fresh Thomson peel (Cserhalmi, Sass‐Kiss, Tóth‐Markus, & Lechner, 2006):

(1) $$\Delta E=\sqrt{{\left(L_{0}-L\right)}^{2}+{\left(a_{0}-a\right)}^{2}+{\left(b_{0}-b\right)}^{2}}$$

Variations in perceptible color, expressed as Δ*E*, can be categorized analytically as not notable (0–0.5), slightly notable (0.5–1.5), notable (1.5–3.0), readily visible (3.0–6.0), and great (6.0–12.0).

Furthermore, browning index (BI) was calculated using measured L*, a*, and b* values according to Equation (2) (Pathare, Opara, & Al‐Said, 2013):

(2) $$\text{BI}=\frac{\left[100\times \left(x-0.31\right)\right]}{0.17}$$

Where

(3) $$x=\frac{\left(a^{*}+1.75\times L^{*}\right)}{\left(5.645\times L^{*}+a^{*}-3.012\times b^{*}\right)}$$

### 2.4 Essential oils analysis

#### 2.4.1 Essential oil extraction

For the intention of extracting EOs via hydrodistillation process under optimal operating conditions, 50 g of Thomson peels was added to 150 ml distilled water in a 500‐ml glass boiling bottle. Next, the clevenger apparatus was placed in a balloon heater and the set was attached to a cold water flow with a view to ensure condensation of EOs. Extraction process was carried out up to 3 hr which after completion, two phases were observed, an organic yellowish phase (EO) with lower density than water and an aqueous phase (aromatic water). Eventually, the EOs were collected, dried under anhydrous sodium sulfate and stored in sealed vials in the dark at 4°C till analysis. Experiments were conducted three times for each treatment. The extraction yield of Thomson peel EOs was calculated according to Equation (4), and it was expressed as ml/g sample (Farahmandfar, Asnaashari, Pourshayegan, Maghsoudi, & Moniri, 2018):

(4) $$\text{EO Yield}\;\%=\left(\text{Volume of extracted EO ml}/\text{Total weight of the sample}\;g\right)\times 100$$

#### 2.4.2 GC‐MS volatile compounds determination

A gas chromatograph (Model 7890A), fitted with an HP‐5 capillary column (0.25 mm i.d. × 30 m length × 25 mm film thickness) coupled to a 5975C mass selective detector quadrupole (Agilent Technologies), was utilized for GC‐MS analysis. The conditions for GC‐MS analysis were in the following terms: helium as carrier gas with a flow rate of 1 ml/min, injection volume of 1 μl, injection temperature of 240°C, split ratio of 70:1, a temperature program, starting at 60°C, hold for 2 min, then increased at 5°C/min till reached 220°C, and ionization energy of 70 eV. The identification of EO constituents was performed by comparison of their kovats retention indices (RI) and retention times (RT) with National Institute of Standards and Technology (NIST 11.0) mass‐spectral libraries and previous literatures. The kovats retention indices were determined using the Dool (1963) equation (Equation 5), and a homologous series of n‐alkanes (C8–C18) injected under the chromatography conditions described above. The quantitative analysis of EO compounds, expressed in percentage, was carried out via the normalization method of the FID peak areas as indicated by Zhang, Chen, Wang, and Yao (2006).

(5) $$\text{RI}=100n_{0}+100\left(\frac{\text{RT}x-\text{RT}n_{0}}{\text{RT}n_{1}-\text{RT}n_{0}}\right)$$

where RI is retention index, *x* is the target compound, *n*_0_ is n‐alkane directly eluting before *x*, *n*_1_ is n‐alkane directly eluting after *x* and RT is the retention time.

#### 2.4.3 Determination of total phenolic content

Total phenol content (TPC) was evaluated by using the method of Farahmandfar et al. (2018). Briefly, 15 μL of appropriately diluted sample was mixed with 75 μL of Folin‐Ciocalteu reagent and 1,185 μL of distilled water in a falcon tube, and after standing for 2–3 min at room temperature, 225 μL of sodium carbonate solution (20%) was added. After incubation of the mixture at room temperature for 20 min in the dark, the absorbance was then read at 750 nm using a spectrophotometer (T80+, PG Instruments Ltd.). Gallic acid was utilized for standard calibration, and the amount of total phenolic was displayed as milligrams of gallic acid equivalents (GAE)/ 100 g sample.

#### 2.4.4 Determination of DPPH radical scavenging activity

This method is based on the measurement of the scavenging ability of antioxidants toward the stable radical DPPH (1,1‐diphenyl‐2‐picrylhydrazyl), and it was conducted according to Farahmandfar, Asnaashari, and Bakhshandeh (2019) with slight modification. Briefly, 2 ml of various dilutions of the test samples was mixed with 2 ml of a 100 μM methanolic DPPH solution. The solution kept for 20 min in the dark, and then its absorbance was measured at 517 nm using a spectrophotometer (T80+, PG Instruments Ltd.). Inhibition of free radical DPPH was calculated using following equation:

(6) $$\text{Inhibition}\left(\%\right)=\left(\frac{A_{\text{blank}}-A_{\text{sample}}}{A_{\text{blank}}}\right)\times 100$$

where *A*_sample_ is the absorbance of the solution when the EOs have been added at different concentrations and *A*_blank_ is the absorbance of the DPPH solution. Likewise, IC50 values of treatments which denote the required concentration of a sample to scavenge 50% of DPPH free radicals, were measured.

#### 2.4.5 Determination of ferric reducing antioxidant potential

The Ferric reducing antioxidant potential (FRAP) of the EOs was measured according to the method of Maurya and Devasagayam (2010). Sample solution (2.5 ml) at different concentrations was combined with 2.5 ml of 200 mmol/L sodium phosphate buffer (pH 6.6) and 2.5 ml of 1% potassium ferricyanide. Then, the mixture was incubated at 50°C for 20 min. After that, 2.5 ml of 10% trichloroacetic acid (w/v) was added to the mixture and then was centrifuged at 3,000 rpm for 8 min (HERMEL Z 9 200A centrifuge). Five milliliters of the upper layer was mixed with 5 ml of deionized water and 1 ml of 0.1% ferric chloride. Finally, the absorbance values of the solutions were read spectrophotometrically at 700 nm. The solution with the higher absorbance value shows higher reducing potential.

#### 2.4.6 Determination of minimum inhibitory concentration and minimum bactericide concentration

The antimicrobial activity of Thomson peel EOs was examined against two gram‐negative bacterial strains (*Pseudomonas aeruginosa* ATCC 9,027 and *Escherichia coli* ATCC 35,218) and two gram‐positive bacterial strains (*Staphylococcus aureus* ATCC 25,904 and *Listeria monocytogenes* ATCC 19,115). Bacterial strains were suspended in ringer solution until the turbidity equal to 0.5 McFarland (1.5 × 10^8^ CFU/ml). The MIC value was determined by the protocols of National Committee for Clinical Laboratory Standards (NCCLS). The broth microdilution method was done using a sterilized 96‐well plate and triphenyl tetrazolium chloride indicator. Microplates containing serial dilutions of peel EOs (0.62, 1.25, 2.5, 5, 10, 20, 40, 80 mg/ml) were prepared, and each well was inoculated with bacterial strains to yield the appropriate density in 100 μl Mueller‐Hinton broth. Then, the 96‐well plate was incubated at 37°C for 24 hr. Minimum inhibitory concentration (MIC) was reported as the lowest concentration of EOs which bacterial growth did not occur and no visible changes being detected in the broth medium (Behbahani, Shahidi, Yazdi, Mortazavi, & Mohebbi, 2017). In relation to minimum bactericidal concentration (MBC) evaluation, all wells in which microbial growth was not observed (opacity‐free), were cultured on Mueller‐Hinton agar and incubated at 37°C for 24 hr. MBC was the lowest concentration in which an antimicrobial agent would kill a particular microorganism (Humeera et al., 2013).

### 2.5 Statistical analysis

Experiments were performed in triplicates, and the results are expressed as means ± standard deviation. Results were subjected to one‐way analysis of variance (ANOVA) using the SPSS version 24.0 software (SPSS Inc.). Statistical significance of differences between samples was accepted at *p* < .05 using the Duncan's multiple range test. In addition, a correlation analysis between total phenolic content and each of the antioxidant capacity assays was performed with the Pearson's test.

## 3 RESULTS AND DISCUSSION

### 3.1 Physical properties

#### 3.1.1 Bulk density

Bulk density is an important indicator of transport cost and packaging considerations. A dry product with high bulk density can be stored in smaller containers than a similar product with lower density (Razavi & Farahmandfar, 2008). Bulk density is well correlated with particle size of dried powders, where smaller powder particles would reduce the porosity and enhance the coherence of dried sample which would lead to a denser product (Sogi, Garg, & Bawa, 2002). According to Table 1, the amount of bulk density in Thomson peel dried powders varied from 1.79 to 2.69 g/ml, highest belonged to MW 360 W and OD 45°C samples and the lowest to FD sample. A similar result in the study of Michalska, Wojdyło, Lech, Łysiak, and Figiel (2016) was observed where MW and OD samples had higher bulk density than other treatments. It was explained that in case of MW, high temperature would cause partial carbonizing of the product, which results in a higher particle/air ratio expressed as bulk density. As far as OD is concerned, application of hot air would cause a considerable shrinkage and collapse of the cell walls which would induce tensions in cellular structure, causing a decline in cell size, roundness, compactness and an increase in elongation (Karam, Petit, Zimmer, Djantou, & Scher, 2016). Though, there was no significant different between bulk densities of OD 45°C and VOD 45°C samples (*p* > .05), applying vacuum in higher drying temperature (60°C) created product with 5.5% lower bulk density than OD 60°C sample (Table 1). Vacuum would allow faster moisture transfer to the surrounding of material and would prevent structural collapse. This process, well‐known as the puffing phenomenon, engenders a porous texture, and thereby it could reduce the material's density (Chong, Figiel, Law, & Wojdyło, 2014). The content of bulk density (1.79 g/ml) in FD powder was the lowest observed among the samples (Table 1). FD would cause the ice in the fresh sample to sublime directly to vapor, which protect the primary structure and shape with minimal shrinkage. Generally, there is a negative correlation between drying temperature and the bulk density as the temperate raises bulk density would reduce (Karam et al., 2016). That was the case for MW as the power increased from 360 to 600 W, bulk density decreased from 2.69 to 2.53 g/ml (Table 1). Similar results were obtained by Horuz and Maskan (2015), which they clarified that, increment in microwave power generally reduces the bulk density of dried products through enhancement of puffing. However, that was not the case in OD and VOD treatments where increase in temperature (from 45 to 60°C) enhanced the bulk density up to 8.09% and 4.54%, respectively. This indicates that higher temperatures not always reduces the density of a material, in some cases depending on plant physiology it would lead to higher shrinkage and cell expansion and eventually a denser product (Argyropoulos, Heindl, & Müller, 2011).

**Table 1:** Bulk density and color values of dried and fresh samples of Thomson peel

	Bulk density (g/ml)	Color measurement				
		*L*	*a*	*b*	Δ*E*	BI
SD	2.39 ± 0.03^c^	41.24 ± 4.72^cd^	8.86 ± 0.58^b^	42.94 ± 2.91^c^	23.46 ± 3.84^bcd^	244.40 ± 21.95^abc^
ShD	2.10 ± 0.08^d^	37.84 ± 1.23^cd^	7.09 ± 0.61^c^	40.50 ± 1.37^cd^	27.78 ± 2.63^bc^	254.58 ± 10.62^ab^
OD 45	2.47 ± 0.05^bc^	38.17 ± 1.24^c^	5.60 ± 0.39^e^	40.80 ± 0.84^c^	28.16 ± 2.42^b^	251.59 ± 8.49^b^
OD 60	2.67 ± 0.03^a^	40.03 ± 1.63^c^	8.61 ± 0.35^b^	41.67 ± 1.05^c^	25.06 ± 0.40^c^	242.22 ± 9.26^b^
VOD 45	2.42 ± 0.03^c^	30.36 ± 0.67^e^	7.93 ± 0.80^bc^	31.04 ± 0.88^e^	38.41 ± 1.42^a^	236.10 ± 5.55^bc^
VOD 60	2.53 ± 0.03^b^	28.36 ± 0.96^f^	8.13 ± 0.60^bc^	30.28 ± 0.80^e^	40.16 ± 1.38^a^	260.79 ± 32.56^abc^
MW 360	2.69 ± 0.03^a^	37.57 ± 1.77^cd^	5.84 ± 0.61^de^	39.71 ± 1.10^cd^	29.05 ± 2.74^b^	246.36 ± 11.98^b^
MW 600	2.53 ± 0.04^b^	35.37 ± 1.49^d^	7.03 ± 0.71^cd^	38.91 ± 1.00^d^	30.32 ± 1.40^b^	271.14 ± 10.24^a^
FD	1.79 ± 0.03^e^	48.54 ± 0.69^b^	1.79 ± 0.37^f^	49.00 ± 1.10^b^	22.04 ± 2.38^d^	216.11 ± 16.64^c^
Fresh	–	53.99 ± 0.91^a^	20.95 ± 1.86^a^	58.31 ± 1.10^a^	–	270.34 ± 14.64^ab^

Note

All values are expressed as mean ± standard deviation. Means (*n* = 3) having different letters within the same column differ significantly at *p* < .05.

Abbreviations: –, not determined; BI, Browning index; SD, Sun drying; ShD, Shade drying; OD 45 and 60, Oven drying at 45°C and 60°C; VOD 45 and 60, Vacuum oven drying at 45°C and 60°C; MW 360 and 600, Microwave drying at 360 W and 600 W; FD, Freeze‐drying.

#### 3.1.2 Color

**Figure 1:**
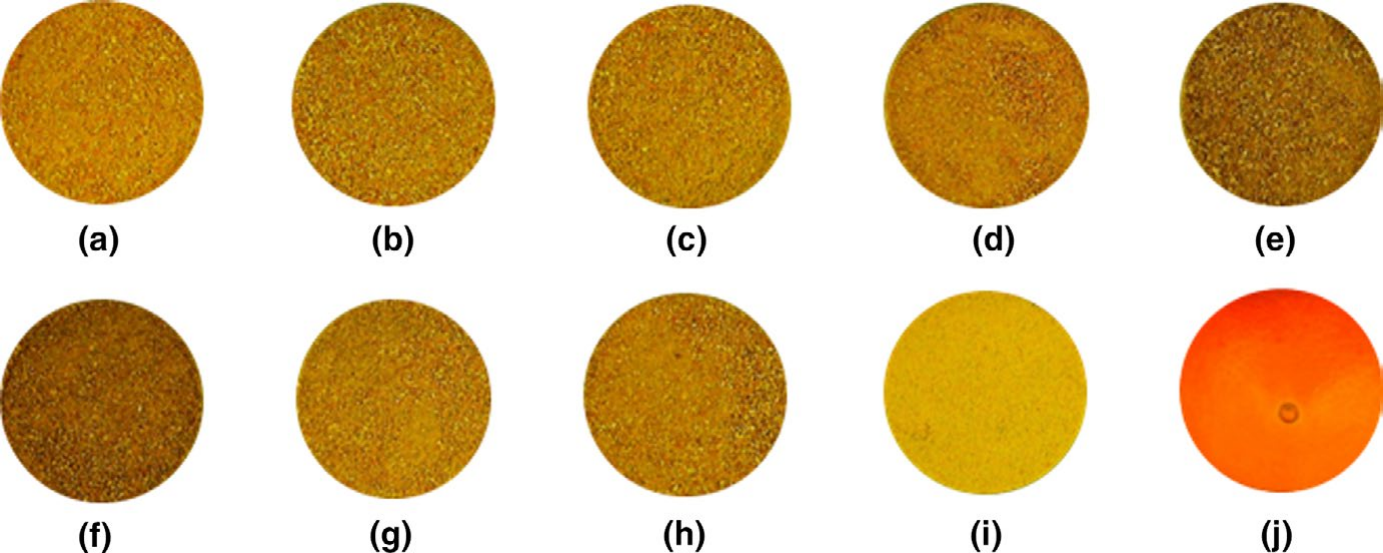
Appearance of Thomson peel samples: (a) sun drying; (b) shade drying; (c) oven drying 45°C; (d) oven drying 60°C; (e) vacuum oven drying 45°C; (f) vacuum oven drying 60°C; (g) microwave drying 360 W; (h) microwave drying 600 W; (i) freeze‐drying and (j) fresh

Color is a critical quality index which influences consumer reception and the market value of the dried materials (M’hiri, Ghali, Nasr, & Boudhrioua, 2018). The color parameters (L*, a*, b*, Δ*E* and BI) and the appearance alterations of dried fruit peels are presented in Table 1 and Figure 1, respectively. The chromatic parameters L* (darkness/brightness), a* (greenness/redness), and b* (blueness/yellowness) values of fresh Thomson peel were 53.99, 20.95, and 58.31, respectively. All color values of all dried samples declined considerably in comparison with the fresh Thomson peel (Table 1). Among dried samples, VOD at 60°C was the darkest with lowest yellowness and highest Δ*E* value which is in agreement with results of Zhang, Liu, and Gao (2018), where least amount of L*, b*, and Δ*E* values were observed in VOD sample. This could be due to the low vacuum pressure of the operation, which caused longer drying times and as a result of exposing to relatively high temperature for a long period of time, higher color deterioration occurred in this method (Arslan, Özcan, & Mengeş, 2010). On the other hand, FD was the best option to preserve majority of color values with the highest lightness (L* = 48.54), yellowness (b* = 49.00) and lowest redness (a* = 1.79). This was in accordance with the findings of Zhang et al. (2018), where FD had the highest content of lightness and yellowness along with lowest redness in *Angelica keiskei* compared with other drying procedures. They acknowledged that FD is the most appropriate technique for retention of fruits and vegetables color quality due to its exceptionally low drying temperature which could preserve pigments responsible for the yellow color like carotenoids and flavonoids after thermal treatment (Ghanem, Mihoubi, Kechaou, & Mihoubi, 2012). Δ*E* is another color index, which shows the degree of overall color change in dried samples compared with the color of fresh Thomson peel. The last color parameter is BI which shows the purity of brown color and is reported as an important parameter in processes where enzymatic and nonenzymatic browning takes place (Saricoban & Yilmaz, 2010). Highest BI belonged to MW 600 W sample (271.14), which may be due to nonenzymatic Maillard browning, and formation of brown pigments at higher drying temperature of MW treatment (Bal, Kar, Satya, & Naik, 2011). FD sample indicated lowest BI value (216.11) which proved that it is the best drying treatment in terms of minimal color deterioration. It should be noted that BI content of fresh material was comparable with all dried powders (*p* > .05) with the exception of FD sample. Fresh Thomson peel contains high amount of moisture which due to its high availability it could promote enzymatic degradation and it might lead to enhance brown color in fresh material (Chua, Chong, Chua, & Figiel, 2019).

### 3.2 Essential oils characteristics

#### 3.2.1 Essential oil yield

**Figure 2:**
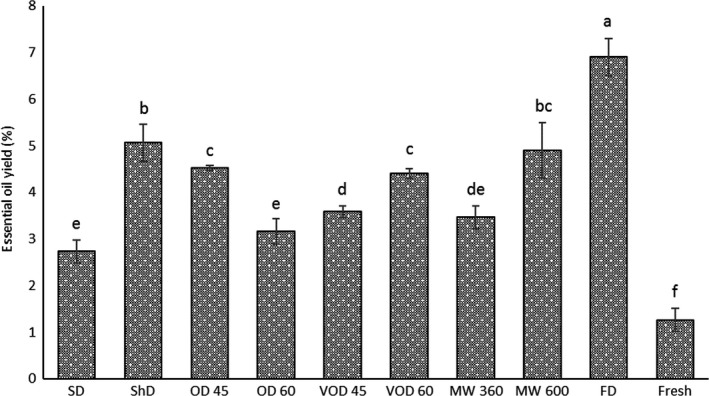
Effect of different drying methods on the essential oil yield of Thomson peels. The vertical bars represent standard deviation (±SD) of the means. SD, Sun drying; ShD, Shade drying; OD 45 and 60, Oven drying at 45°C and 60°C; VOD 45 and 60, Vacuum oven drying at 45°C and 60°C; MW 360 and 600, Microwave drying at 360 W and 600 W; FD, Freeze‐drying

The results showed that various drying methods notably effects the EO content of Thomson peel (Figure 2) and depending on the type of drying method, duration, and temperature both increment and reduction in the quantities of EO yields were observed (Rahimmalek & Goli, 2013). The highest EO yield (6.90% v/w) obtained via FD whereas the lowest EO yield (1.20% v/w) was noticed in fresh sample. These results were in agreement with the study of Rahimmalek and Goli (2013) where FD sample of *Thymus daenensis* subsp. *daenensis* had the highest EO yield compared with other drying techniques, and it can be explained by the FD temperature which is the lowest among drying treatments. Thus, it could maintain more EOs in the dried sample and preserve aromatic compounds from diffusion into the atmosphere (Rahimmalek & Goli, 2013). As far as fresh sample is concerned, the lowest extraction yield could be due to its large particle size. All dried samples were ground and screened by a standard sieve; however, fresh sample merely got chopped to small pieces and due to its high moisture content grinding process was not possible as a sticky paste would be formed. Accordingly, it has been acknowledged in previous studies that there is a negative correlation between particle size and extraction efficiency where in larger particle size, solvent diffusion in solid material would decrease due to low surface to volume ratio and it would reduce EO extraction (Eikani, Golmohammad, & Rowshanzamir, 2007). Moreover, increasing the OD temperature (from 45 to 60°C) significantly decreased the EO content (Figure 2). This would prove that drying temperature increment in OD would cause severe loss in EOs extraction, and it could be due to multiple impediments that come with oven drying, including vast ventilation where a large volume of air flowing through samples over a long duration allows volatile compounds to easily evaporate, in addition to creating an environment for high oxidation to occur (Chua et al., 2019; Figiel, Szumny, Gutiérrez‐Ortíz, & Carbonell‐Barrachina, 2010). Similar results on the higher loss of volatiles were also observed for the OD samples of oregano (Figiel et al., 2010) and *Dracocephalum kotschyi Boiss*. (Samadi et al., 2018). It was also reported in previous studies that temperature enhancement for VOD and MW treatments would cause higher rates of reduction in the EO content of some plant species such as *Dracocephalum kotschyi Boiss*. (Samadi et al., 2018), peppermint leaves (Salarikia, Miraei Ashtiani, & Golzarian, 2017), and *Mentha longifolia* L. (Saeidi, Ghafari, & Rostami, 2016). However, in our study, increasing temperature in VOD and MW methods enhanced the EO content of dried samples. This proves that the effects of higher drying temperature could be variable in different plants, and it might be emanated from physiological differences in plant species, secretory tissue, their localization and chemical composition of EO (Rahimmalek & Goli, 2013; Salarikia et al., 2017).

#### 3.2.2 GC‐MS compounds identification

In the current experiment, 39 components were identified in the EOs of dried and fresh Thomson peel samples representing 93.3%–100.2% of the total volatile oils (Table 2). The volatile compounds of essential oils could be categorized in the following main chemical groups: monoterpene hydrocarbons (83.55%–93.31%), oxygenated monoterpenes (5.70%–8.97%), and sesquiterpenes (0.63%–2.83%). In fresh Thomson peel, the major components of the EOs were limonene (71.54), β‐myrcene (7.20%), linalool (4.11%), α‐pinene (1.85%), sabinene (1.70%), and decanal (1.21%). As stated in previous reports, EO constituents of Thomson peel could be considerably influenced by intrinsic (genetics, subspecies, and plant age) or extrinsic (geographical origin, climate condition, and isolation methods) factors (Duman, Soltanbeigi, & Ozcan, 2016; Kirbaslar et al., 2009; Nekoei & Mohammadhosseini, 2014; Njoroge et al., 2009; Xiao, Ma, Niu, Chen, & Yu, 2016; Yang et al., 2017). By way of illustration, the quantity of major volatile compounds of Turkish Thomson peel EO in the study of Duman et al. (2016) were as follows: limonene (71.80%), β‐myrcene (4.55%), sabinene (1.39%), linalool (3.89%), and α‐pinene (1.17%), which were significantly different compared with present study.

**Table 2:** Contents of volatile compounds in Thomson peel essential oils

No.	Volatile compounds	RT (min)	RI	Pick area (%)									
				SD	ShD	OD 45	OD 60	VOD 45	VOD 60	MW 360	MW 600	FD	Fresh
*Monoterpenes*													
1	***α*‐Pinene**	5.64	–	2.42^b^	2.48^b^	3.53^a^	2.58^b^	2.62^b^	2.39^b^	2.66^b^	2.74^b^	1.26^d^	1.85^c^
2	*β*‐Phellandrene	6.67	1,093	0.63^cd^	0.88^bc^	0.51^d^	0.37^e^	1.83^a^	0.74^c^	0.76^c^	0.70^c^	1.08^b^	0.72^c^
3	**Sabinene**	6.74	1,000	1.32^b^	1.72^ab^	1.20^b^	1.03^c^	1.30^b^	1.38^abc^	1.40^ab^	1.79^a^	0.80^d^	1.70^ab^
4	***β*‐Myrcene**	7.23	1,025	8.69^a^	7.33^b^	6.62^c^	8.07^a^	5.54^d^	6.78^bc^	7.91^ab^	8.19^a^	4.91^d^	7.20^b^
5	**Limonene**	8.72	1,081	79.00^bc^	79.58^bc^	76.34^d^	78.45^c^	80.03^b^	78.06^c^	79.86^bc^	79.42^bc^	81.88^a^	71.54^e^
6	*β*‐Ocimene	9.14	1,200	0.16^ab^	0.19^ab^	–	0.21^a^	0.16^ab^	0.18^a^	0.16^ab^	0.18^ab^	0.12^b^	0.16^ab^
7	*γ*‐Terpinene	9.35	1,091	0.31^b^	0.18^c^	0.32^b^	0.47^a^	0.29^b^	0.25^b^	0.17^c^	0.12^d^	0.15^cd^	0.23^bc^
8	*p*‐Menthene	10.05	855	0.18^cd^	0.16^d^	0.70^a^	0.28^b^	0.23^bc^	0.21^bc^	0.22^bc^	0.17^cd^	0.14^d^	0.15^cd^
	Total			92.71^b^	92.52^b^	89.22^c^	91.46^abc^	92.00^abc^	89.99^bc^	93.14^a^	93.31^a^	89.62^b^	83.55^d^
*Oxygenated Monoterpenes*													
9	1‐Octanol	9.58	1,031	0.07^e^	0.09^e^	0.20^d^	0.23^cd^	0.50^b^	0.26^c^	0.27^c^	0.26^c^	0.24^cd^	0.92^a^
10	**Linalool**	10.43	1,084	1.89^d^	2.06^d^	2.79^b^	1.61^e^	1.09^g^	1.32^f^	1.28^f^	1.63^e^	2.37^c^	4.11^a^
11	Nonanal	10.50	1,007	0.30^a^	0.30^a^	–	0.30^a^	0.22^b^	0.30^a^	0.30^a^	0.31^a^	0.31^a^	–
12	*trans*‐Limonene oxide	11.42	982	–	0.06^b^	0.11^a^	–	–	–	–	–	–	–
13	*β*‐Terpineol	11.61	1,045	–	–	–	0.10^a^	–	–	0.09^a^	–	–	–
14	Citronellal	11.84	1,042	0.11^b^	0.16^a^	0.20^a^	–	–	0.11^b^	0.07^c^	0.12^b^	–	0.07^c^
15	Isoneral	12.15	1,058	–	–	0.05^a^	–	–	–	–	–	–	–
16	1‐Nonanol	12.37	1,100	–	–	–	–	0.07^a^	–	–	–	–	–
17	Terpinen‐4‐ol	12.57	934	0.83^b^	0.38^d^	0.46^c^	0.89^a^	0.62^b^	0.42^cd^	0.38^d^	0.24^e^	0.27^e^	0.38^d^
18	*α*‐Terpineol	12.95	1,048	0.73^b^	0.63^c^	0.89^a^	0.87^a^	0.64^c^	0.49^d^	0.85^a^	0.48^d^	0.82^a^	0.85^a^
19	**Decanal**	13.36	1,053	1.01^e^	1.12^d^	1.48^b^	1.23^c^	1.21^c^	1.49^b^	1.21^c^	1.22^c^	1.80^a^	1.21^c^
20	*cis*‐Carveol	13.71	1,057	0.10^b^	0.08^b^	0.15^a^	–	0.11^ab^	–	–	–	–	–
21	Nerol	13.97	1,041	–	–	0.14^b^	0.16^b^	0.30^a^	0.17^b^	0.10^c^	0.10^c^	–	0.10^c^
22	Neral	14.33	1,080	0.45^b^	0.42^bc^	0.63^a^	0.28^d^	0.12^e^	0.30^d^	0.37^c^	0.42^bc^	0.55^a^	0.37^c^
23	Carvone	14.42	1,025	–	–	0.06^a^	–	–	–	–	–	–	–
24	Geraniol	14.69	1,036	–	–	0.06^c^	–	0.27^a^	0.12^b^	0.07^c^	–	–	0.07^c^
25	Geranial	15.15	1,085	0.63^c^	0.60^c^	0.83^a^	0.44^e^	0.32^f^	0.55^cd^	0.53^d^	0.59^c^	0.73^b^	0.53^d^
26	Phellandral	15.23	1,008	0.11^b^	0.10^b^	0.15^a^	0.08^c^	–	0.08^c^	0.10^b^	0.09^bc^	–	0.10^b^
27	Undecanal	16.09	1,031	–	–	0.10^a^	–	0.08^ab^	0.11^a^	0.06^b^	0.06^b^	–	0.06^b^
28	Dodecanal	18.74	1554	0.13^e^	0.16^de^	0.28^b^	0.21^c^	0.26^b^	0.35^a^	0.20^cd^	0.18^c^	0.39^a^	0.20^c^
	Total			6.36^c^	6.16^cd^	8.58^a^	6.40^c^	5.81^cd^	6.07^cd^	5.88^cd^	5.70^d^	7.48^b^	8.97^a^
*Sesquiterpenes*													
29	*α*‐Copaene	17.93	1,183	0.09^d^	0.10^d^	0.19^b^	0.14^c^	0.18^b^	0.24^a^	0.12^cd^	0.11^cd^	0.20^b^	0.12^cd^
30	*β*‐Cubebene	18.30	1588	–	0.07^b^	0.15^a^	–	0.08^b^	0.15^a^	0.01^c^	0.02^c^	0.17^a^	0.01^c^
31	*β*‐Elemene	18.35	1511	–	0.07^b^	0.13^a^	0.09^b^	0.09^b^	0.14^a^	–	–	–	–
32	*β*‐Caryophyllene	19.06	1,257	0.10^d^	0.12^d^	0.20^b^	0.16^c^	0.16^c^	0.23^a^	0.10^d^	0.11^d^	0.24^a^	0.10^d^
33	*β*‐Copaene	19.30	1527	0.06^e^	0.07^e^	0.15^b^	0.10^d^	0.13^c^	0.18^a^	0.09^de^	0.09^d^	0.16^ab^	0.09^de^
34	*cis‐β*‐Farnesene	19.94	1549	–	–	0.12^b^	0.11^b^	0.10^b^	0.16^a^	–	–	–	–
35	Germacrene	20.59	1568	0.08^e^	0.10^de^	0.18^b^	0.12^d^	0.14^c^	0.22^a^	0.09^e^	0.10^de^	0.20^ab^	0.09^de^
36	Valencene	20.89	1549	–	–	0.40^a^	0.25^b^	0.26^b^	0.36^a^	0.14^c^	0.17^c^	0.36^a^	0.14^c^
37	*α*‐Farnesene	21.20	1542	–	–	0.12^b^	0.11^b^	0.10^b^	0.16^a^	–	–	–	–
38	*Δ*‐Cadinene	21.62	1505	0.17^cd^	0.17^cd^	0.29^b^	0.26^b^	0.27^b^	0.43^a^	0.18^c^	0.15^d^	0.38^a^	0.18^cd^
39	*β*‐Sinensal	28.87	–	0.13^f^	0.21^e^	0.34^c^	0.30^cd^	0.28^d^	0.56^b^	0.14^f^	0.13^f^	0.69^a^	0.14^f^
	Total			0.63^e^	0.91^d^	2.27^b^	1.64^c^	1.79^c^	2.83^a^	0.87^d^	0.88^d^	2.40^ab^	0.87^d^
Total			99.70^a^	99.59^a^	100.07^a^	99.50^a^	99.60^a^	98.89^a^	99.89^a^	99.89^a^	99.50^a^	93.39^b^	

Note

Means (*n* = 3) having different letters within the same row differ significantly at *p* < .05. Major volatile components are shown in bold text.

Abbreviations: –, not detected; RT, Retention time; RI, Calculated retention index; SD, Sun drying; ShD, Shade drying; OD 45 and 60, Oven drying at 45°C and 60°C; VOD 45 and 60, Vacuum oven drying at 45°C and 60°C; MW 360 and 600, Microwave drying at 360 W and 600 W; FD, Freeze‐drying.

As shown in Table 2, different drying methods had notable effects on all major components identified in the EOs. Among the active ingredient groups, monoterpene hydrocarbons were the most important ones as they possessed the majority of main volatile components. Although the utmost content of monoterpene hydrocarbons was obtained by MW 600 W (93.31%), different components of this group were changed differently when using other drying methods (Table 2). The highest amount of limonene (81.88%), β‐myrcene (8.69%), and α‐pinene (3.53%) was noticed in FD, SD, and OD 45°C, respectively. In comparison with other drying treatments, VOD 45°C led to more contents of β‐phellandrene (1.83%). The highest amounts of β‐ocimene (0.21%) and γ‐terpinene (0.47%) was measured for OD 60°C, though the maximum amounts of p‐menthene (0.70%) and sabinene were obtained by OD 45°C and MW 600 W, respectively. Overall, the total amount of monoterpene hydrocarbons in dried samples (Table 2) were higher than fresh sample (*p* < .05). Primarily, monoterpene hydrocarbons are categorized as nonpolar compounds and it seems that these compounds have low affinity to the water fraction of fruit peels. Thereby, they would not be evaporated along with water during hydrodistillation process (Hazrati, Farnia, Habibzadeh, & Mollaei, 2018). On the other hand, drying processes can make a porous structure on plant materials and help availability of solutes, for example, essential oil, via increment of mass transfer coefficient during extraction, thus higher volatile compounds than fresh sample can be obtained (Feyzi, Eikani, Golmohammad, & Tafaghodinia, 2017). This was not in agreement with the results of prior work done by Samadi et al. (2018) and Pirbalouti, Mahdad, and Craker (2013) where dried samples of *Dracocephalum kotschyi Boiss.* and basil landrace, respectively, had lower amount of monoterpene hydrocarbons than fresh sample. The differences in the outcomes might be related to the type and origin of the plants as well as the conditions used for drying treatments (Shahhoseini, Estaji, Hosseini, Ghorbanpour, & Omidbaigi, 2013). Moreover, the changes in the oxygenated monoterpenes contents (specifically linalool and decanal as the major oxygenated monoterpenes) are displayed in Table 2. Although the highest content of linalool (4.11%) was obtained was observed in fresh sample, FD procedure showed higher content of decanal (1.80%) compared with other treatments. As indicated in Table 2, not merely drying processes triggered the elimination of some oxygenated monoterpenes and sesquiterpenes, likewise they caused the appearance of other volatile compounds which were absent in fresh peel EO. For instance, some components such as citronellal, nerol, geraniol, phellandral, undecanal, *β*‐cubebene, and valencene were present in fresh peels while they have been disappeared in several dried samples (Table 2), whereas nonanal, *trans*‐limonene oxide, *β*‐terpineol, isoneral, 1‐nonanol, *cis*‐carveol, carvone, *β*‐elemene, *cis‐β*‐farnesene, and *α*‐farnesene were absent in fresh fruit peels but they appeared in several dried samples. Similar findings were reported by researchers (Rahimmalek & Goli, 2013; Samadi et al., 2018), which they clarified that this phenomenon may be attributed to the formation or removal of volatile compounds by esterification, oxidation, glycoside hydrolysis, and other processes during drying. The total amount of sesquiterpenes was influenced by the drying treatments as well. VOD sample at 60°C led to more amount of sesquiterpenes than other drying procedures. As is shown in Table 2, the highest amounts of β‐sinensal (0.69%) and Δ‐cadinene (0.43%) (as the main sesquiterpenes of EO) were determined in samples dried by FD and VOD (60°C) methods, respectively. Overall, sesquiterpenes have higher molecular weight than monoterpenes, and thus, they are less volatile and hardly removed from the plant material; however, they are susceptible to oxidation reaction and exposing the samples for extended drying times would reduce the sesquiterpene compounds (Chua et al., 2019). That could explain the highest retention of these components by VOD 60°C where oxygen is minimized and in contrast the greatest loss of these compounds by SD which has the highest drying time in atmospheric condition. Similar results were observed in the study of Samadi et al. (2018) where sun drying had the highest sesquiterpenes loss, while VOD at 50°C treatment was among the best methods of preserving sesquiterpenes.

#### 3.2.3 Total phenol content

**Figure 3:**
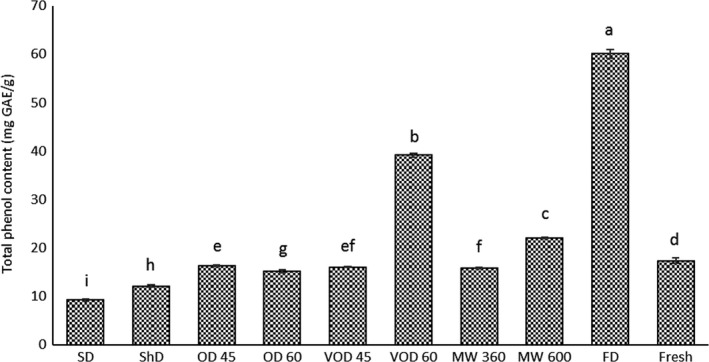
Effects of different drying methods on total phenol content of Thomson peel essential oils. The vertical bars represent standard deviation (±SD) of the means. SD, Sun drying; ShD, Shade drying; OD 45 and 60, Oven drying at 45°C and 60°C; VOD 45 and 60, Vacuum oven drying at 45°C and 60°C; MW 360 and 600, Microwave drying at 360 W and 600 W; FD, Freeze‐drying

TPC of Thomson peel EOs were shown in Figure 3. As demonstrated, the amount of TPC in SD, ShD, OD (45 and 60°C) MW (360 W), and VOD (45°C) samples were lower than their fresh counterpart. In case of SD and ShD, usually their lower drying temperature could be beneficial in preserving heat‐labile antioxidant compounds; however, unexpected precipitation and cloud condition could influence the drying rate and due to extended drying duration, increase in enzymatic degradation could occur (Chua et al., 2019). Similar reduction in TPC of SD and ShD samples was observed in ginger, tomato (Gümüşay, Borazan, Ercal, & Demirkol, 2015), *Vitex negundo* and *Vitex trifolia* (Chong & Lim, 2012). Concerning to OD samples the lower TPC could be the result of the high oxidation process which has taken place during the prolong exposure of samples to hot air (Chong & Lim, 2012). In the study of Yi and Wetzstein (2011), exposure to 70°C OD caused highest TPC loss in rosemary, motherwort and peppermint leaves. Furthermore, the TPC of other drying treatments were as following order: FD > VOD 60°C > MW 600W which the amounts of TPC in all of them were higher than fresh sample (*p* < .05). There are three explanations for this situation. First of all, highest TPC of FD sample is due to lower drying temperature of FD process where utmost retention of heat‐sensitive compounds is attainable (Chong & Lim, 2012). This was asserted in previous studies where FD samples showed highest TPC content in ginger (Gümüşay et al., 2015), *Vitex negundo* and *Vitex trifolia* (Chong & Lim, 2012). Secondly, when plants are under drying treatments, their tissues are more fragile and higher temperatures in MW or VOD would cause an easier breakdown of cell walls which would boost the extractability of antioxidants during extraction (Hossain, Barry‐Ryan, Martin‐Diana, & Brunton, 2010). On the other hand, total drying time would be reduced and samples would be less subjected to destructive effects of high drying temperatures (Chua et al., 2019). Thirdly, lower TPC in fresh sample could be due to its high moisture content, which promotes enzymatic reaction and that may result in the loss of antioxidant compounds (Chua et al., 2019; Hossain et al., 2010).

#### 3.2.4 Antioxidant activity

In this study, the antioxidant activity of Thomson peel EOs was investigated by two distinct methods: DPPH˙ scavenging test and ferric reducing/antioxidant power assay. In the DPPH test, the stable free radical with dark violet color interact with the phenolic compounds and immediately after receiving proton from them, it instantly loses its chromophore and becomes yellow (Farahmandfar et al., 2018; İnan, Özcan, & Aljuhaimi, 2018). Therefore, the discoloration degree would indicate the potentials of free radical scavenging of the analyzed compound (Sayyad & Farahmandfar, 2017). Likewise, reducing potential of antioxidants is an important indicator of their activity, and it could be measured through FRAP test which evaluates the reduction in ferric ion (Fe^3+^)–ligand complex to the intensely blue colored ferrous (Fe^2+^) complex by antioxidants in acidic media (Shahidi & Zhong, 2015). Antioxidant activities (DPPH and FRAP tests) of Thomson peel EOs at concentrations of 5000–80,000 ppm are given in Tables 3 and 4, respectively. In both experiments, with increase in concentrations of the EOs, the scavenging of free radicals and reducing power of these compounds increased, which is due to the increasing amount of phenolic compounds at higher concentrations of the EOs, similar to the results of previously published studies of researchers (Farahmandfar et al., 2018; Sayyad & Farahmandfar, 2017). As indicated in Table 3, different drying methods with varied conditions would significantly influence the DPPH scavenging activity of EOs. In DPPH test, the highest antioxidant activity (lowest IC50) was observed in FD sample, followed by VOD 60°C, MW 600W, fresh, VOD 45°C, OD 45°C, MW 360W, ShD, OD 60°C, and SD samples (Table 3). We found a quite strong negative correlation (Table 5) between total phenol content and IC50 concentration (*R* ^2^ = 0.704), which proved that higher phenolic compounds would enhance scavenging activity (reduce IC50) of Thomson peel EOs, as FD, VOD 60°C, and MW 600 W samples with highest TPCs showed highest antioxidant activities (lowest IC50) and SD sample with lowest TPC had lowest scavenging activity (highest IC50). Similar results were observed in the prior published work of An et al. (2016) on Chinese ginger where the DPPH scavenging ability had high correlation with TPC (*R* ^2^ = 0.866), with highest TPC and lowest IC50 belonged to FD sample.

**Table 3:** DPPH radical scavenging activities of Thomson peel essential oils

Treatments	Inhibition (%)					IC 50 (mg/ml)
	5 mg/ml	10 mg/ml	20 mg/ml	40 mg/ml	80 mg/ml	
SD	30.30 ± 0.52^i^	39.40 ± 0.52^i^	50.00 ± 0.50^i^	56.96 ± 0.25^g^	59.06 ± 0.40^i^	20.00^a^
ShD	35.46 ± 0.59^h^	48.43 ± 0.51^e^	51.30 ± 0.36^h^	67.90 ± 0.55^c^	70.46 ± 0.45^c^	15.04^c^
OD 45	40.66 ± 0.41^e^	47.36 ± 0.40^f^	58.00 ± 0.40^e^	62.33 ± 0.35^e^	68.36 ± 0.35^e^	14.23^e^
OD 60	37.20 ± 0.25^g^	41.20 ± 0.20^h^	52.14 ± 0.22^g^	54.53 ± 0.47^h^	65.30 ± 0.36^g^	16.07^b^
VOD 45	43.30 ± 0.36^d^	50.51 ± 0.41^d^	65.03 ± 0.04^c^	70.10 ± 0.10^b^	78.16 ± 0.20^b^	7.99^f^
VOD 60	47.20 ± 1.02^b^	56.30 ± 0.36^a^	67.30 ± 0.26^b^	69.53 ± 0.45^b^	78.73 ± 0.37^b^	7.24^i^
MW 360	39.30 ± 0.26^f^	45.20 ± 0.45^g^	56.36 ± 0.15^f^	59.43 ± 0.73^f^	67.60 ± 0.45^f^	14.76^d^
MW 600	45.60 ± 0.40^c^	54.36 ± 0.30^b^	60.23 ± 1.07^d^	63.56 ± 0.11^d^	69.50 ± 0.55^d^	7.50^h^
FD	50.00 ± 1.20^a^	56.19 ± 0.26^a^	68.90 ± 1.41^a^	74.20 ± 0.30^a^	87.90 ± 0.36^a^	5.00 ^j^
Fresh	42.73 ± 0.75^d^	52.66 ± 0.87^c^	58.10 ± 0.55^e^	62.40 ± 0.52^e^	64.26 ± 0.25^h^	7.86^g^

Note

All values are expressed as mean ± standard deviation. Means (*n* = 3) having different letters within the same column differ significantly at *p* < .05.

Abbreviations: SD, Sun drying; ShD, Shade drying; OD 45 and 60, Oven drying at 45°C and 60°C; VOD 45 and 60, Vacuum oven drying at 45°C and 60°C; MW 360 and 600, Microwave drying at 360 W and 600 W; FD, Freeze‐drying.

**Table 4:** Ferric reducing/antioxidant power of Thomson peel essential oil

Treatments	Inhibition (%)				
	5 mg/ml	10 mg/ml	20 mg/ml	40 mg/ml	80 mg/ml
SD	0.03 ± 0.00^gh^	0.05 ± 0.01^f^	0.07 ± 0.01^e^	0.15 ± 0.00^g^	0.23 ± 0.00^g^
ShD	0.01 ± 0.00^h^	0.16 ± 0.00^d^	0.21 ± 0.00^c^	0.27 ± 0.00 ^f^	0.32 ± 0.01^f^
OD 45	0.09 ± 0.00^f^	0.16 ± 0.02^d^	0.21 ± 0.01^c^	0.33 ± 0.01^c^	0.38 ± 0.01^d^
OD 60	0.04 ± 0.01^g^	0.08 ± 0.01^e^	0.15 ± 0.01^d^	0.29 ± 0.01^ef^	0.35 ± 0.01^e^
VOD 45	0.12 ± 0.01^e^	0.18 ± 0.01^cd^	0.23 ± 0.02^c^	0.3 ± 0.00^de^	0.41 ± 0.02^c^
VOD 60	0.22 ± 0.01^b^	0.26 ± 0.06^b^	0.30 ± 0.01^b^	0.36 ± 0.01^b^	0.48 ± 0.03^b^
MW 360	0.03 ± 0.01^gh^	0.16 ± 0.02^d^	0.23 ± 0.01^c^	0.31 ± 0.01^cd^	0.36 ± 0.01^de^
MW 600	0.09 ± 0.00^f^	0.16 ± 0.02^d^	0.21 ± 0.01^c^	0.33 ± 0.01^c^	0.38 ± 0.01^d^
FD	0.21 ± 0.01^ab^	0.25 ± 0.02 ^b^	0.34 ± 0.08 ^ab^	0.37 ± 0.01 ^a^	0.52 ± 0.12 ^a^
Fresh	0.15 ± 0.01^d^	0.20 ± 0.00^c^	0.22 ± 0.01^c^	0.31 ± 0.01^cde^	0.42 ± 0.02^c^

Note

All values are expressed as mean ± standard deviation. Means (*n* = 3) having different letters within the same column differ significantly at *p* < .05.

Abbreviations: SD, Sun drying; ShD, Shade drying; OD 45 and 60, Oven drying at 45°C and 60°C; VOD 45 and 60, Vacuum oven drying at 45°C and 60°C; MW 360 and 600, Microwave drying at 360 W and 600 W; FD, Freeze‐drying.

**Table 5:** Correlation analysis between the measured antioxidant parameters of the experiment

Parameters	TPC	DPPH (IC50)	FRAP (80 mg/ml)
TPC	1.000		
DPPH (IC50)	−0.704*	1.000	
FRAP (80 mg/ml)	0.836**	−0.897**	1.000

Abbreviations: TPC, total phenol content, DPPH, 2, 2‐diphenyl‐1‐picrylhydrazyl assay; FRAP, ferric reducing antioxidant power assay.

* Indicate significant correlation at the 0.05 level.

** Indicate significant correlation at the 0.01 level.

The results of FRAP test was quite similar to DPPH essay, since there was a relatively strong correlation between TPC and FRAP value (*R* ^2^ = 0.836), as well (Table 5). At the highest EO concentration (80,000 ppm) FD was the dominant drying technique in respect of reducing activity due to its higher phenolic content, on the other hand, SD sample with lowest TPC was merely the weakest treatment with regard to FRAP value. This was in accordance to the study of Gümüşay et al. (2015) on tomato and ginger, where a similar trend was observed, as FD and SD had the highest and lowest reducing capacity compared with other drying treatments. Effect of temperature raise in FRAP method was not as clear as DPPH test, since there was no significant difference between microwave treatments (360 and 600W) (*p* > .05); however, much like the DPPH assay, a slight increase and decrease in FRAP value of VOD and OD samples was observed respectively, as temperature raised from 45 to 60°C, which is undoubtedly related to their phenolic contents.

#### 3.2.5 Antibacterial activity

The MICs and MBCs of Thomson peel EOs against gram‐negative and gram‐positive bacteria are reported in Table 6. As illustrated, different drying treatments showed various degrees of activity against the tested strains. All EOs were more effective against gram‐positive bacteria than gram‐negative bacteria in the present study. This was in agreement with previous studies of researchers (Burt, 2004; Geraci et al., 2017), and this can be attributed to diversities in their cell structure. The outer peptidoglycan layer in gram‐negative bacteria is an ineffective permeability barrier, since the prions present in these types of bacteria would limit the entry of solutes and make them less susceptible to antibacterial components (Singh, Negi, & Radha, 2013). According to Table 6, *S. aureus* and *E.* *coli* were the most sensitive tested strains and the variation in MIC and MBC values were more clarified in these bacteria than *L. monocytogenes* and *P. aeruginosa*. The best treatment in terms of antibacterial activities was FD followed by VOD at 60°C and MW at 600 W which the amount of MIC and MBC values were much lower than fresh sample, in contrast the weakest ones (highest MIC and MBC values) were SD and ShD (Table 6). It was reported in prior studies that antibacterial activity is well correlated with the amount of phenolic compounds within Eos (Singh et al., 2013), specifically by virtue of containing the most well‐known and characterized compounds of the citrus EOs such as limonene, α‐pinene, sabinene, β‐myrcene, and linalool which due to their synergistic effect, they can apply a strong and vast spectrum of antimicrobial activities. However, since these compounds are very hydrophobic and are difficult to disperse in water, high concentrations must be applied in order for them to be effective antimicrobial components (Calo, Crandall, O'Bryan, & Ricke, 2015). That is why, FD, VOD (60°C) and MW (600 W) samples with higher TPC (Figure 3) had higher antimicrobial activities and contrarily SD and ShD samples with lower TPC showed feeble antibacterial activities compared to other treatments.

**Table 6:** Antimicrobial activity of the Thomson navel orange essential oils against selected bacterial strains

Treatments	Test microorganisms							
	Gram‐negative				Gram‐positive			
	*Escherichia coli*		*Pseudomonas aeruginosa*		*Staphylococcus aureus*		*Listeria monocytogenes*	
	MIC	MBC	MIC	MBC	MIC	MBC	MIC	MBC
SD	20	40	80<	80<	5	10	80<	80<
ShD	10	20	80<	80<	5	20	80<	80<
OD 45	5	20	80	80<	2.50	5	40	40
OD 60	5	10	80<	80<	2.50	10	80<	80<
VOD 45	10	20	80<	80<	2.50	5	40	80
VOD 60	1.25	2.50	20	80	0.62	1.25	10	20
MW 360	2.50	10	80<	80<	2.50	10	80	80
MW 600	1.25	2.50	20	40	1.25	2.50	10	20
FD	1.25	1.25	10	20	0.31	0.62	5	10
Fresh	5	20	40	80<	2.50	5	20	40

Abbreviations: FD, Freeze‐drying; MBC, minimum bactericidal concentration (μg/mL); MIC, minimum inhibitory concentration (μg/mL); MW 360 and 600, Microwave drying at 360 W and 600 W; OD 45 and 60, Oven drying at 45°C and 60°C; SD, Sun drying; ShD, Shade drying; VOD 45 and 60, Vacuum oven drying at 45°C and 60°C.

## 4 CONCLUSION

The current study is the first to report the effects of six drying methods with varied conditions on the physical and EO properties of Thomson peel. In summary, highest amount of color lightness and yellowness as well as lowest content of ΔE and BI was observed in FD sample. The EO extracted via FD sample had higher values of extraction yield, limonene (the major volatile compound of Thomson peel EO), TPC, DPPH scavenging, reducing power along with lowest MIC and MBC against four bacteria strains compared with other drying treatments of this study. Our findings could provide a valuable data base for developing a process for dehydration of Thomson peel, and FD could be the potential method for producing an excellent dry product with highest quality EOs.

## CONFLICT OF INTEREST

The authors declare that they do not have any conflict of interest.

## ETHICAL APPROVAL

This study does not involve any human or animal testing.
